# Does the *in vitro* egg hatch test predict the failure of benzimidazole treatment in *Haemonchus contortus*?

**DOI:** 10.1051/parasite/2021059

**Published:** 2021-08-18

**Authors:** Michal Babják, Alžbeta Königová, Michaela Urda Dolinská, Tomas Kupčinskas, Jaroslav Vadlejch, Georg von Samson-Himmelstjerna, Saulius Petkevičius, Marián Várady

**Affiliations:** 1 Institute of Parasitology, Slovak Academy of Sciences Hlinkova 3 040 01 Košice Slovakia; 2 Lithuanian University of Health Sciences Tilzes 18 47181 Kaunas Lithuania; 3 Department of Zoology and Fisheries, Faculty of Agrobiology, Food and Natural Resources, Czech University of Life Sciences Prague Kamýcká 129 165 00 Prague Suchdol Czech Republic; 4 Freie Universität Berlin, Institute for Parasitology and Tropical Veterinary Medicine Robert von Ostertag Str. 7-13 14163 Berlin Germany

**Keywords:** Anthelmintic resistance, Detection methods, Egg hatch test, *Haemonchus contortus*, Goats

## Abstract

Considerable research has been directed towards optimising *in vitro* tests that can diagnose resistance in pre-parasitic stages of parasites. The objective of this study was to compare the *in vivo* faecal egg count reduction test (FECRT), the *in vitro* egg hatch test (EHT), and the molecular determination of the frequency of a codon 200 allele of β-tubulin isotype 1 associated with benzimidazole resistance in larval stages of *Haemonchus contortus* obtained from infected goats. Animals were infected with composite infective doses representing 10, 20, 30, 40, 60, and 80% resistant alleles. Faecal samples for the EHT were collected on 28, 33, and 35 days post-infection. The results of the *in vivo* FECRT indicated that albendazole treatment reduced infections consisting of composite doses of 10, 20, 30, 40, 60, and 80% larvae of the resistant isolate by 91.3, 78.0, 63.3, 48.4, 36.5, and 41.4%, respectively. The drug concentration at which 50% of the eggs were prevented from developing hatching larvae (ED_50_) in the *in vitro* EHT varied from 0.09 ± 0.01 to 15.63 ± 12.10 μg/mL thiabendazole. The results of the *in vitro* EHT indicated that the test could estimate *in vivo* resistance well. The EHT could thus accurately estimate the *in vivo* efficacy of the drug and percentage of the resistance allele in the population using hatching parameters in delineation doses. This finding was also supported by comparing the FECRT data to the hatching percentages in the EHT on 30 goat farms in Slovakia with natural mixed infections of gastrointestinal parasites.

## Abstract (fr)

Des efforts de recherche considérables ont été consacrés à l’optimisation des tests *in vitro* permettant de diagnostiquer la résistance des parasites aux stades préparasitaires. L’objectif de cette étude était de comparer le test de réduction du nombre d’œufs fécaux *in vivo* (TRNOF), le test d’éclosion des œufs *in vitro* (TEO) et la détermination moléculaire de la fréquence d’un allèle du codon 200 de l’isotype 1 de la β-tubuline associé à résistance au benzimidazole au stade larvaire d’*Haemonchus contortus*, obtenus à partir de chèvres infectées. Les animaux ont été infectés avec des doses infectieuses composites représentant 10, 20, 30, 40, 60 et 80 % d’allèles résistants. Des échantillons de matières fécales ont été prélevés pour le TEO aux jours 28, 33 et 35 après l’infection. Les résultats de la TRNOF *in vivo* ont indiqué que le traitement à l’albendazole réduisait respectivement de 91,3, 78,0, 63,3, 48,4, 36,5 et 41,4 % les infections composées de doses composites de 10, 20, 30, 40, 60 et 80 % de larves de l’isolat résistant. La concentration de médicament à laquelle 50 % des œufs ont été empêchés de développer des larves qui éclosent (DE50) dans le TEO *in vitro* variait de 0,09 ± 0,01 à 15,63 ± 12,10 μg/mL de thiabendazole. Les résultats du TEO *in vitro* ont indiqué que le test pouvait correctement estimer la résistance *in vivo*. Le TEO a ainsi pu estimer avec précision l’efficacité *in vivo* du médicament et le pourcentage de l’allèle de résistance dans la population en utilisant des paramètres d’éclosion dans les doses de délimitation. Cette constatation a également été étayée par la comparaison des données du TRNOF aux pourcentages d’éclosion dans le TEO sur 30 élevages de chèvres en Slovaquie, avec des infections mixtes naturelles de parasites gastro-intestinaux.

## Introduction

The efficient use of anthelmintics is an integral part of worm control strategies for preventing production losses from parasitic infections. The intensive and/or inappropriate use of anthelmintics, however, leads to the development of anthelmintic resistance (AR), which has become important in most countries with intensive productions of small and large ruminants [33]. AR in most countries is characterised by widespread benzimidazole (BZ) resistance. Some countries, mostly with Mediterranean climates, however, still have a low prevalence of BZ resistance [1, 8, 13, 14, 23, 25]. The accurate diagnosis of resistance in these countries would help to prevent AR from becoming widespread.

The growing importance of AR has led to an increased need for reliable and standardised methods of detection. Several methods are currently being used to detect AR. The faecal egg count reduction test (FECRT), which is predictive for all anthelmintic classes, has long been used as the gold standard. Various *in vitro* tests measuring the effects of anthelmintics on physiological processes such as development, growth, and movement have been developed as alternatives for detecting AR. The egg hatch test (EHT) and larval development test are the most frequently used *in vitro* methods for detection of BZ resistance. Both *in vitro* tests have demonstrated comparable and reliable results for detecting BZ resistance and have an advantage over other techniques due to their higher sensitivity in identifying relatively small proportions (4%) of resistant worms in a population [28]. The EHT has been described in various studies [5–7, 18, 31] and is based on the ovicidal activity of BZs, forming the basis for many other *in vitro* tests. The main disadvantage of the FECRT and EHT is that they can only detect resistance when >25% of a nematode population contains the resistance allele [20].

The sensitivity of the EHT can be substantially enhanced using a discriminating dose rather than ED_50_ [28]. The EHT has been standardised in ring testing in several laboratories using previously identified laboratory isolates susceptible and resistant to anthelmintics [30]. Validation of current *in vitro* tests or simple, sensitive, and reliable new field tests with strong correlations to the *in vivo* FECR test, however, are needed.

The main objective of this study was to answer three questions: (i) How is the correlation between BZ resistance defined phenotypically and genotypically? (ii) Does BZ resistance confirmed by *in vitro* testing correspond to *in vivo* FEC percentage reduction? and (iii) Are the results of the *in vitro* EHT and the *in vivo* FECRT correlated in field surveys?

## Material and methods

### Ethical considerations

All procedures and animals were cared for under European Community guidelines (EU Directive 2010/63/EU). The experimental protocol was approved by the Ethics Committee of the Institute of Parasitology of the Slovak Academy of Sciences (protocol code 2018/14), following national legislation in Slovakia (G.R. 377/2012; Law 39/2007) for the care and use of research animals. Permission to collect samples and to carry out the experiment was granted by the participating goat farmers.

### Parasite isolates

Two isolates of *Haemonchus contortus* were used, one susceptible and one resistant to BZs. The susceptible isolate, ISE, was obtained as an inbred isolate of MHCo3 [26]. The resistant isolate was isolated from a sheep farm at the University of Georgia, Athens, USA. This isolate had been passaged in goats since 2007. The frequency of the BZ resistance associated allele at codon 200 of β-tubulin isotype 1 in each isolate was established using a Pyrosequencing™ assay [29]. The percentages of the TAC resistance allele at codon 200 were 86% in the resistant isolate and 4% in the susceptible isolate. Infective L3 for the composite doses for genotyping were obtained by larval culture using standard techniques (MAFF, 1986). Different proportions of larvae from the resistant and susceptible isolates were mixed to prepare infective composite doses consisting of larvae representing 10, 20, 30, 40, 60, and 80% resistant alleles.

### Trial design

Fifty-four Saanen goat kids were randomly allocated into six groups of nine animals. The goats were 3–4 months old with initial body weights of approximately 15 kg and were housed in common stalls for seven days for adjusting to feeding, with free access to water. The goats in each group were infected with 2500 third-stage (L3) larvae from each of the composite doses. Faecal samples for *in vitro* EHT were collected 28, 33, and 35 days post-infection and stored in anaerobic conditions until laboratory examination. A modified McMaster technique [6] with a sensitivity of 50 eggs per gram (EPG) of faeces was used to detect strongylid eggs. The FECRT was conducted following WAAVP recommendations [6, 7]. The goats were treated with the dose of albendazole (ABZ) recommended for ovines (5 mg/kg body weight) at day 35 post-infection. The animals in each group were resampled eight days after treatment. The percent reduction in helminth eggs (%FECR) was calculated using the method of individually based estimation of Cabaret et al. [4]:

$$\%\mathrm{FECR}=(1/n)\sum (100\times (1-[T_{i2}/T_{i1}]),$$

where *T*_*i*1_ and *T*_*i*2_ are pre- and post-treatment EPGs, respectively, for host *i* from a total of *n* hosts. The parasite population was considered resistant if %FECR was *<*95% [4]. Pooled faecal samples for larval culture were collected before treatment from each group on day 35 post-infection.

### *In vitro* EHT

Nematode eggs for all composite doses were collected by sequentially sieving through three stacked sieves with apertures of 250, 100, and 25 μm. The material retained on the 25-μm sieve was washed with water, sedimented, floated in a saturated solution of sodium chloride [6], collected on a 20-μm sieve, and washed with water; the eggs were subsequently used for the *in vitro* EHT as described by Coles et al. [6]. A stock solution of thiabendazole (TBZ) (Sigma-Aldrich Chemie GmbH, Taufkirchen, Germany) was prepared by dissolving the pure compound in dimethylsulphoxide (DMSO). Final concentrations in each well of 24-well plates (TPP Techno Plastic Products AG, Trasadingen, Switzerland) were prepared by adding 10 μL of each TBZ solution (or DMSO for control wells) to 1.99 mL of an aqueous suspension of approximately 150 eggs/mL. The final TBZ concentrations were 0.05, 0.1, 0.3, 0.5, and 1 μg/mL. A control (0.5% DMSO) without anthelmintic was also included in the test. Plates were incubated at 27.0 °C for 48 h. The incubation was then terminated by adding 10 mL of Lugol’s iodine to each well. The test was performed with two replicates for each drug concentration on six occasions (six independent days) for each composite dose. Discriminating a dose of 0.1 mg/mL thiabendazole was used as the cut-off criterion for the presence of resistance [7].

### Pyrosequencing assay to determine the frequencies of β-tubulin alleles

Genomic DNA isolated from a pool of approximately 5000 *H. contortus* L3 larvae obtained from each group of goats on day 35 post-infection was used as the template for PCR for each composite dose. DNA extraction and pyrosequencing assays targeting the F200Y codon of the isotype-1 β-tubulin gene have previously been described and were used in our study employing Hc200PySeq1 (5k – TAG AGA ACA CCG ATG AAA CAT – 3k) as the sequencing primer at 0.4 μμM [29].

### Statistical analysis

Data from a field survey of the prevalence of BZ resistance in Slovakia [2] were used to correlate the results of the *in vitro* EHT and *in vivo* FECRT. The FECRT results from 30 goat farms in this comparison using formulae of individually based estimation of FECR proposed by Cabaret et al. [4] were correlated with the hatching percentage in EHTs conducted on these farms. The data from the EHTs were analysed using a statistical logistic regression model [11] to determine the drug concentration at which half of the eggs were prevented from hatching (ED_50_). A linear regression analysis was carried out to correlate the proportion of resistance alleles, %FECR (dependent variables), and ED_50_, hatching percentages (independent variables) obtained by the EHT (GraphPad Prism 8, GraphPad Software, Inc., San Diego, CA, USA).

## Results

The arithmetic mean and minimum and maximum %FECR in the groups of goat kids infected with the different doses of the resistant isolate are presented in Table 1. The results of the FECRT, taking into account the resistance threshold, indicated that all groups resistant to ABZ were detected. The pyrosequencing results for codon 200 are also presented in Table 1. The percentages of the TAC allele of β-tubulin isotype 1 were very similar with the estimated percentages of resistant larvae in the infective doses in each group of goats. Importantly, the results of the FECRT were comparable with the genotypes of the L3 larvae derived from the worms used to infect the animals. These results confirmed the tendency that a higher percentage of resistance alleles led to lower effectiveness of BZ in all groups.

**Table 1:** Percent faecal egg count reduction (%FECR) and percentage of the codon 200 TAC allele of β-tubulin isotype 1 in the groups of goats infected with different proportions of the resistant and susceptible isolates of *Haemonchus contortus*.

Calculated % of R alleles in infection dose	%FECR	%FECR (min–max)	TAC (%)*
10	91.2	75.0–97.3	10.5
20	77.9	77.8–100.0	22.8
30	63.1	27.3–87.3	39.5
40	50.5	0–85.8	40.0
60	36.5	0–88.6	53.5
80	41.3	0–69.1	74.0

* Larvae obtained from pooled culture from each group before treatment at 35 days post-infection.

Table 2 presents the percentages of hatching at the four highest concentrations of TBZ. The percentage of eggs hatching at 0.1, 0.3, 0.5, and 1.0 μg/mL TBZ for the groups of animals ranged from 36.3 to 93.3, 13.6 to 89.7, 7.0 to 87.7, and 4.8 to 84.7%, respectively. These results indicated the presence of resistant parasites in all groups. ED_50_ values ranged from 0.09 to 15.63 μg/mL. Resistance was confirmed in the groups infected with >30% resistant larvae using a threshold of 0.1 μg/mL. The statistical correlations between the tests are presented in Table 3. The regressions examining the feasibility of using the results of one test to predict the results of another test were significant (*p* < 0.05) in eight of 10 cases. The results of the correlations indicated that the phenotypic expression of BZ resistance by hatching in the EHT can accurately predict the efficacy in the FECRT or the percentage of resistance alleles identified by pyrosequencing. ED_50_ in the EHT and FECRT, however, was weakly correlated with the pyrosequencing results. The correlations between the results of the FECRT and hatching (%) in the EHT in the field survey of goat farms [2] are presented in Table 4. The coefficients of determination were generally high and significant (*p* < 0.05) in all cases.

**Table 2:** Mean hatching (%) at different thiabendazole (TBZ) concentrations and ED_50_ in the EHT in the groups of goats infected with different proportions of the resistant and susceptible isolates of *Haemonchus contortus*.

Calculated % of R alleles in infection dose	Hatching (%) ± SD at TBZ concentration (μg/mL)				
	0.1	0.3	0.5	1.0	ED_50_ (μg/mL TBZ)
10	36.3 ± 13.3	12.6 ± 5.3	7.0 ± 2.9	4.8 ± 2.4	0.09 ± 0.01
20	35.7 ± 9.2	28.7 ± 6.6	24.8 ± 5.3	14.8 ± 6.0	0.1 ± 0.01
30	60.2 ± 8.3	46.8 ± 10.6	46.4 ± 11.4	34.8 ± 6.7	0.36 ± 0.09
40	75.9 ± 6.1	62.1 ± 11.1	55.9 ± 7.9	45.8 ± 10.2	0.72 ± 0.31
60	76.2 ± 4.8	71.4 ± 7.6	65.3 ± 7.2	59.7 ± 7.3	1.88 ± 0.44
80	93.3 ± 2.7	89.7 ± 4.1	87.7 ± 4.1	84.7 ± 5.2	15.63 ± 12.10

**Table 3:** Coefficients of determination and *p* values for comparing the results of the faecal egg count reduction test (FECRT), percentage hatching in the egg hatch test (EHT) at the four highest concentrations of thiabendazole (μg/mL), frequency (%) of the rr genotype, and ED_50_ in the EHT in the experimental infections with *Haemonchus contortus*.

Comparison	Coefficient of determination	*P*
FECRT vs. EHT hatching at 0.1	0.872	0.0063
FECRT vs. EHT hatching at 0.3	0.925	0.0021
FECRT vs. EHT hatching at 0.5	0.897	0.0041
FECRT vs. EHT hatching at 1.0	0.860	0.0077
rr vs. EHT hatching at 0.1	0.890	0.0047
rr vs. EHT hatching at 0.3	0.956	0.0007
rr vs. EHT hatching at 0.5	0.979	0.0002
rr vs. EHT hatching at 1.0	0.982	0.0001
FECRT vs. EHT ED_50_	0.264	0.296
rr vs. ED_50_ EHT	0.639	0.056

**Table 4:** Coefficients of determination and *p* values for comparing the results of the faecal egg count reduction test (FECRT) and percentage hatching in the egg hatch test (EHT) at the four highest concentrations of thiabendazole (μg/mL) in a field survey of 30 goat farms.

Comparison	Coefficient of determination	*P*
FECRT^a^ vs. EHT hatching at 0.1	0.416	0.0002
FECRT^a^ vs. EHT hatching at 0.3	0.506	0.001
FECRT^a^ vs. EHT hatching at 0.5	0.567	0.0001
FECRT^a^ vs. EHT hatching at 1.0	0.491	0.0001
FECRT^b^ vs. EHT hatching at 0.1	0.591	0.0001
FECRT^b^ vs. EHT hatching at 0.3	0.724	0.0001
FECRT^b^ vs. EHT hatching at 0.5	0.845	0.0001
FECRT^b^ vs. EHT hatching at 1.0	0.745	0.0001

a FECRT using albendazole at dose of 5 mg/kg body weight.

b FECRT using albendazole at dose of 10 mg/kg body weight.

## Discussion

The EHT has long been used to detect AR, most often in the diagnosis of AR in sheep [1, 10, 12, 17], goats [2, 15, 22], and horses [16, 27, 32]. The test is based on the ovicidal effect of BZ and the ability of eggs to embryonate and hatch at higher BZ concentrations than for susceptible strains [18]. Results are evaluated based on exceeding the ED_50_ cut-off of 0.1 μg/mL (the concentration of anthelmintic required to prevent hatching of 50% of the eggs) established by Coles et al. [6].

A sensitive, fast, and cost-effective “pen side” (field test) method is needed to detect the early stages of resistance in populations due to the rapid spread of AR. Martin et al. [20] reported that the EHT could not detect resistance using the ED_50_ criterion if ≤25% of the population comprised resistant worms, providing the basis for the claim that the EHT cannot confirm resistance when a population contains ≤25% resistant individuals. Pyrosequencing in our study accurately determined the genotypic composition of the L3 larvae obtained from the individual groups of animals infected with varying proportions of the resistant isolate, which certainly contributed to the more accurate results. We also used percent hatching instead of ED_50_ in the EHT as a more sensitive criterion to evaluate resistance [9]. A TBZ concentration of 0.1 μg/mL prevented the hatching of 99% of the susceptible isolate, as suggested by Coles et al. [7]. These authors, however, also suggested that further studies would be needed to determine the percentage of eggs hatching at this concentration as diagnostic of BZ resistance.

The results of *in vitro* tests should provide a precise estimate of *in vivo* efficacy. ED_50_ for groups infected with 10.5 and 22.8% resistance alleles, determined by pyrosequencing ranged from 0.09 to 0.1 μg/mL TBZ. Using ED_50_ would mistakenly classify both groups as sensitive. The percentage of hatching in both groups at a TBZ concentration of 0.1 μg/mL varied from 35.7 to 36.3%. Infection with only 10.5 and 22.8% resistance alleles in groups 1 and 2, respectively, however, suggested that eggs with an sr genotype (heterozygotes) are also likely to hatch at this concentration. Accounting for hatching at a higher TBZ concentration of 0.3, 0.5, or 1.0 μg/mL, which are more similar to the percentages of resistance alleles derived by genotyping, would then be better.

The experimental kids were treated orally with 5 mg ABZ.kg^−1^ body weight, the recommended dose for small ruminants (sheep and goats) or only for sheep in Slovakia. In Slovakia, there is no BZ anthelmintic specifically registered for use only in goats. In Table 4 coefficients of determination between percentage hatching in the EHT and FECRT are consistently higher when ABZ was used at 10 mg/kg body weight. This could indicate that the use of a double dose could better estimate *in vivo* efficacy. However, the aim of this study was to determine whether hatching in the EHT is a good predictor of *in vivo* efficacy in laboratory and field research. This was fully confirmed in experimental infections with *H. contortus* as well as in mixed infections in field conditions. Additionally, the real prevalence of BZ resistance may be underestimated when using a double dose in FECRTs [2]. In our experiment, we decided to use only groups of goats, with different levels of BZ resistance without a fully susceptible reference strain. The main reason was that in our previous study [9], we phenotypically and genotypically characterised 4 susceptible strains of *H. contortus*.

Detecting resistance under field conditions is more complicated due to mixed infections of gastrointestinal nematodes. von Samson-Himmelstjerna et al. [29] suggested lowering the threshold to 0.05 μg/mL TBZ if *H. contortus* was dominant in a sample, indicating the importance of species identification in mixed infections as an important part of diagnosis. ED_50_ on 15 of the 30 goat farms in the field survey [2] was lower than the threshold of 0.1 μg/mL TBZ, but hatching at this concentration ranged from 5.5 to 38.5%. Using the lower threshold of 0.05 μg/mL allowed [2] us to identify an additional 15 farms with low levels of resistance in the nematode populations. The same study found substantial differences in hatching at “resistant concentrations” (≥0.1 μg/mL thiabendazole) on farms with the same ED_50_. Two farms with an ED_50_ of 0.05 had mean hatching percentages of 11.5 and 32% at 0.1 μg/mL TBZ and 11 and 27.5% at 0.3 μg/mL, respectively. Holm et al. [15] similarly reported hatching at a concentration of 0.3 μg/mL TBZ on two goat farms where ED_50_ was <0.1 μg/mL TBZ. These differences were probably due to the different frequencies of resistance and sensitive alleles in the tested populations, due to the different proportions of heterozygous sr worms [3].

Preventive measures focused on slowing down the spread of resistance are easier to apply when the percentage of resistant population is detected early, i.e. at levels of 5–20%, than when the percentage of resistant population has already reached a level of 30–40%. However, at this range, the ED_50_ value would indicate a susceptible population. The results of the *in vivo* FECRT, hatching at the “resistant concentration”, and the pyrosequencing results were strongly correlated in our study. The correlations between %FECR and ED_50_ and between the frequency of rr genotypes and ED_50_, though, were weak. The results from EHTs to FECRTs are not always correlated in field studies, sometimes producing false positives or false negatives [10, 16, 21]. Rialch et al. [24] documented negative correlations between %FECR and ED_50_. Maharshi et al. [19] similarly found a moderate correlation between %FECR and the frequency of the r allele and that the correlation between %FECR and ED_50_ increased when the rr genotype was >30% in the population.

AR in BZ-resistant strains can also be detected using a discriminating dose (ED_99_) [28]. These authors suggested that using ED_99_ in EHTs could substantially increase test sensitivity when only a small proportion of an *H. contortus* population was resistant. The suitability of ED_95_/ED_99_ as a more sensitive indicator has also been discussed in EHT ring testing conducted by [30], where ED_99_ was >0.1 μg/mL TBZ for all resistant isolates. Using this threshold can thus reduce the possibility of false negatives, but a third of the susceptible strains also had an ED_95_ > 0.1 μg/mL, suggesting that using this cut-off in field studies could increase the number of false positives.

## Conclusions

In conclusion, the results of these experimental and field studies confirmed: (i) a strong correlation between genotype and BZ resistance in *H. contortus* defined by hatching in the EHT, (ii) that hatching percentage in the *in vitro* EHT corresponded to *in vivo* FECRT, and (iii) a significant correlation between hatching in the EHT and %FECR derived from a field survey of AR on goat farms. We also confirmed that hatching percentage in the EHT could generally be used to predict the efficacy of BZs in *in vivo* FECRTs or to predict the percentage of resistance alleles in a population.

## Competing interest

The authors have no conflicts of interest to declare.
